# Successful revascularization of chronic total renal artery occlusion in a young patient with resistant hypertension and renal dysfunction: a case report

**DOI:** 10.1093/ehjcr/ytag373

**Published:** 2026-05-26

**Authors:** Dogancan Cenelı, Tezel Kovancı, Kadır Bıyıklı, Nesrı Danısman, Faruk Akalın

**Affiliations:** Kartal Koşuyolu High Specialization Training and Research Hospital, Denizer Caddesi Cevizli Kavşağı No: 2, Cevizli, Kartal 34870, Istanbul, Turkey; Kartal Koşuyolu High Specialization Training and Research Hospital, Denizer Caddesi Cevizli Kavşağı No: 2, Cevizli, Kartal 34870, Istanbul, Turkey; Kartal Koşuyolu High Specialization Training and Research Hospital, Denizer Caddesi Cevizli Kavşağı No: 2, Cevizli, Kartal 34870, Istanbul, Turkey; Kartal Koşuyolu High Specialization Training and Research Hospital, Denizer Caddesi Cevizli Kavşağı No: 2, Cevizli, Kartal 34870, Istanbul, Turkey; Kartal Koşuyolu High Specialization Training and Research Hospital, Denizer Caddesi Cevizli Kavşağı No: 2, Cevizli, Kartal 34870, Istanbul, Turkey

**Keywords:** Renal artery stenosis, Chronic total occlusion, Resistant hypertension, Renal artery revascularization, Ischaemic nephropathy, Case report

## Abstract

**Background:**

Randomized clinical trials have shown no overall advantage of renal artery revascularization over optimal medical therapy in unselected populations with renal artery stenosis. However, these findings may not be generalizable to all patient subgroups. Carefully selected individuals with high-risk features may still derive substantial clinical benefit from revascularization.

**Case summary:**

A 29-year-old woman presented with severe resistant hypertension despite treatment with four antihypertensive agents and strict adherence to lifestyle modifications. Laboratory investigations revealed newly developed renal dysfunction with a significant reduction in estimated glomerular filtration rate compared with prior normal values. Renal Doppler ultrasonography demonstrated chronic total occlusion of the right renal artery, which was subsequently confirmed by computed tomography angiography and invasive renal angiography. Despite complete arterial occlusion, renal size was preserved, and collateral perfusion via the suprarenal artery was evident. Technetium-99 m dimercaptosuccinic acid scintigraphy confirmed viable renal parenchyma, indicating potential reversibility of ischaemic injury. Given persistent uncontrolled blood pressure, progressive deterioration of renal function, and preserved renal viability, percutaneous renal artery revascularization was undertaken. The occluded segment was successfully revascularizated by stent implantation, resulting in complete restoration of arterial patency. Blood pressure normalized promptly after the procedure, allowing significant down-titration of antihypertensive therapy. Renal function improved markedly within 2 weeks and remained stable during long-term follow-up.

**Discussion:**

Chronic total renal artery occlusion does not invariably reflect irreversible renal damage. In selected patients with preserved renal anatomy, collateral circulation, and viable parenchyma, endovascular revascularization may lead to sustained improvements in blood pressure control and renal function.

Learning points

- Resistant hypertension with new-onset renal dysfunction in young patients should immediately raise suspicion for secondary causes, particularly renal artery stenosis.
- Chronic total renal artery occlusion can still benefit from revascularization in carefully selected patients.
- Renal artery revascularization may yield durable clinical improvement in selected high-risk patients despite neutral trial data.

## Introduction

Renal artery stenosis (RAS) refers to a haemodynamically significant narrowing in the arterial lumen, resulting in impaired renal perfusion. Atherosclerosis represents the predominant aetiology in the general population, accounting for approximately 90% of cases. Less common causes include fibromuscular dysplasia and inflammatory vasculitides.^1^ The prevalence of RAS in the general population is estimated to be approximately 5–10% and increases with advancing age, particularly in male patients.^1,2^ RAS is commonly associated with hypertension, hyperlipidaemia, diabetes mellitus, and chronic kidney disease.^3^ In the treatment of RAS, routine revascularization has not been shown to be superior to medical therapy alone with respect to long-term outcomes, including mortality, renal disease progression, and hypertension control.^3,4^ Nevertheless, these findings may not be applicable to all patient populations. We report the case of a 29-year-old patient with chronic total occlusion (CTO) of the renal artery who experienced marked improvement in renal function and successful blood pressure regulation after renal artery revascularization.

## Summary figure

**Figure:**
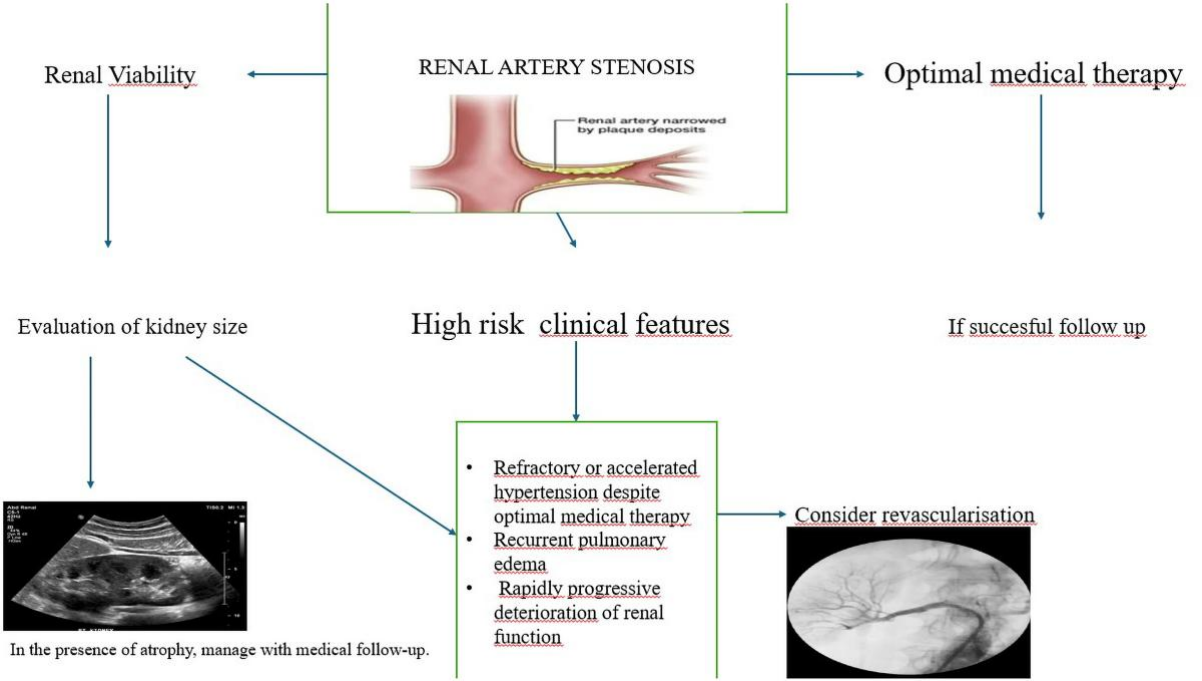


## Case presentation

A 29-year-old female patient presented to our clinic with persistent hypertension despite ongoing antihypertensive therapy. The patient reported good adherence to her prescribed medications and a low-sodium diet. Her past medical history was unremarkable except for a diagnosis of hypertension for the past 2 years. Her current antihypertensive regimen consisted of perindopril 10 mg/day, amlodipine 10 mg/day, hydrochlorothiazide 25 mg/day, and nebivolol 10 mg/day. The patient stated that her systolic blood pressure measurements during 1 month of home monitoring were consistently ≥160 mmHg. On initial evaluation, her vital signs revealed a systolic blood pressure of 168 mmHg, and a diastolic blood pressure of 104 mmHg. Physical examination was unremarkable.

**Figure 1:**
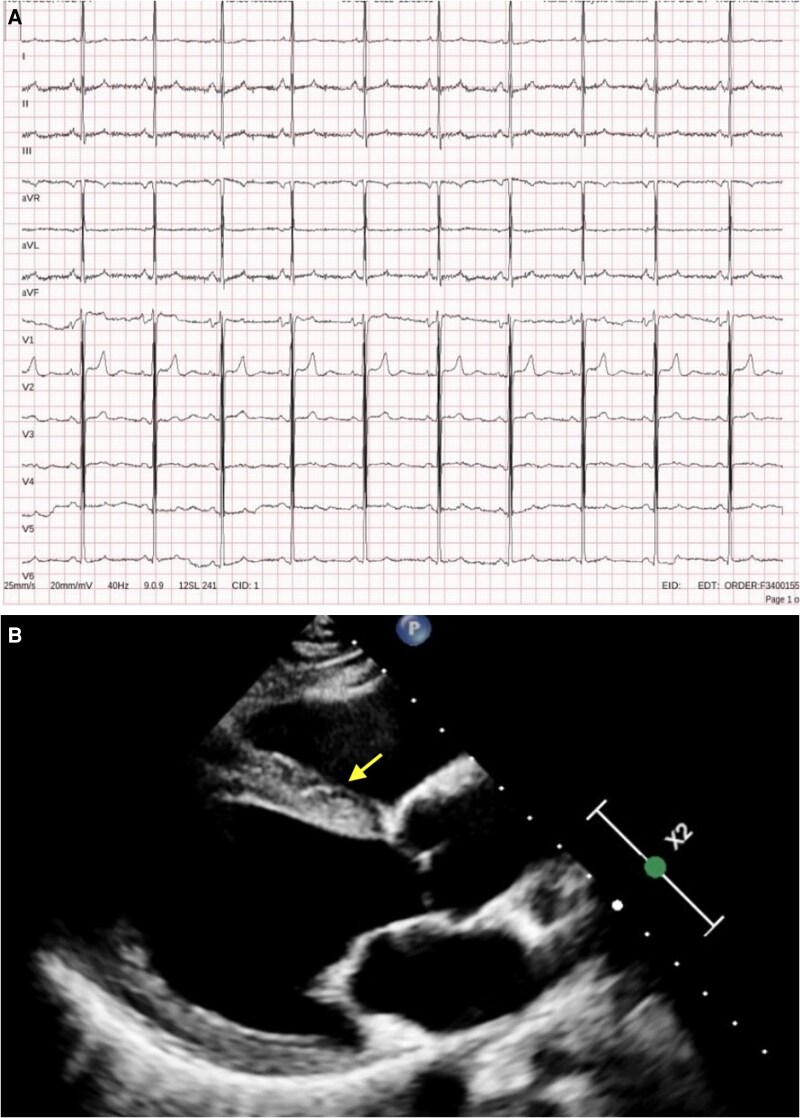
*(A)* The electrocardiogram demonstrates normal sinus rhythm, with findings consistent with concentric left ventricular hypertrophy. *(B)* Transthoracic echocardiography revealed a normal ejection fraction, and concentric left ventricular hypertrophy was identified.

In view of the diagnosis of resistant hypertension, the patient was admitted to our clinic for further evaluation of secondary causes of hypertension. Laboratory evaluation, including endocrine screening and electrolyte assessment, revealed a normal complete blood count, electrolyte levels, and thyroid function tests. Renal function tests demonstrated a urea level of 124 mg/dL and a creatinine level of 2.7 mg/dL, and an estimated glomerular filtration rate of 27 mL/min/1.73 m2. Notably, the patient’s urea and creatinine levels recorded 2 years earlier had been within normal limits. Her electrocardiogram demonstrated normal sinus rhythm with voltage criteria consistent with left ventricular hypertrophy according to the Sokolow–Lyon criteria (*Figure 1A*). Transthoracic echocardiography demonstrated preserved left ventricular systolic function and concentric left ventricular hypertrophy, with an interventricular septal thickness of **1.2 cm** and a posterior wall thickness of **1.3 cm**; the left atrial diameter was **3.7 cm**, the aortic root diameter **3.4 cm**, the tricuspid annular diameter **2.8 cm**, and right ventricular systolic function was preserved with a tricuspid annular plane systolic excursion (TAPSE) of **2.1 cm**, with no significant valvular heart disease (*Figure 1B*).

**Figure 2:**
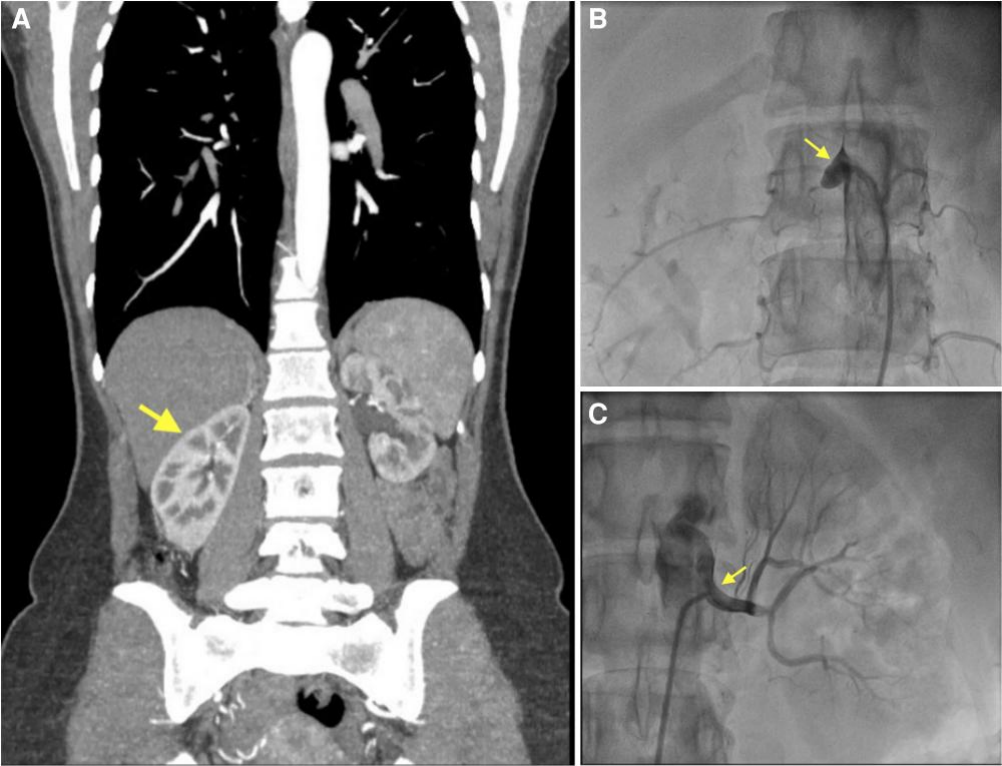
*(A)* Contrast-enhanced computed tomography demonstrated patency of the left renal artery and total occlusion of the right renal artery. Additionally, computed tomography findings confirmed preserved renal size, with no radiological evidence of renal atrophy. *(B)* Conventional angiography demonstrated total occlusion of the right renal artery beginning at its proximal segment. *(C)* Conventional angiography demonstrated patency of the left renal artery.

**Figure 3:**
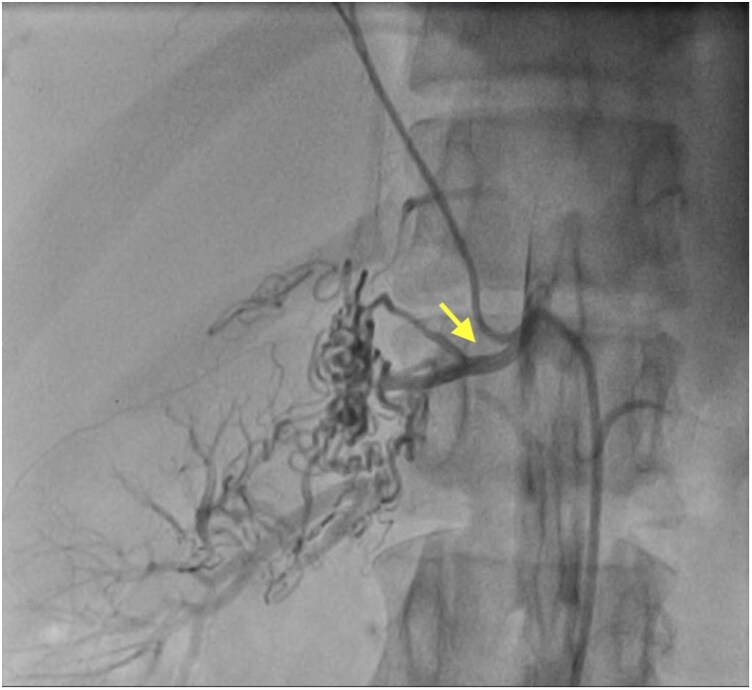
The right kidney was observed to be supplied by collateral vessels originating from the right suprarenal artery.

Given the coexistence of resistant hypertension and impaired renal function, renal ultrasonography and renal Doppler ultrasonography (DUS) were performed to investigate secondary causes. Imaging demonstrated bilaterally preserved renal size (right kidney 104 mm, left kidney 101 mm; normal adult range approximately 90–120 mm) and normal cortical thickness, indicating preserved renal parenchyma. However, DUS revealed occlusion of the right renal artery. In the patient with right renal artery occlusion, further evaluation was performed using subsequent contrast-enhanced computed tomography (CT) and conventional renal angiography confirmed total occlusion of the right renal artery from its proximal segment (*Figure 2A, B, C*; see Video  *S1A*, *B*, *C*). Collateral circulation supplying the right kidney via the suprarenal artery was observed, suggesting that the occlusion was chronic in nature (*Figure 3*; see Video  *S2*). Given the patient’s young age, non-atherosclerotic causes of RAS were considered. Fibromuscular dysplasia was excluded based on the absence of characteristic imaging findings, and inflammatory vasculitides were ruled out through clinical assessment and normal inflammatory markers, supporting an atherosclerotic aetiology. Owing to normal right renal parenchymal measurements and adequate collateral perfusion from the suprarenal artery, Technetium-99 m dimercaptosuccinic acid (Tc-99 m DMSA) scintigraphy was performed and confirmed viable right renal parenchyma. Due to persistent resistant hypertension despite intensive medical therapy, progressive deterioration of renal function, and the presence of viable renal parenchyma, revascularization of the right renal artery was planned.

**Figure 4:**
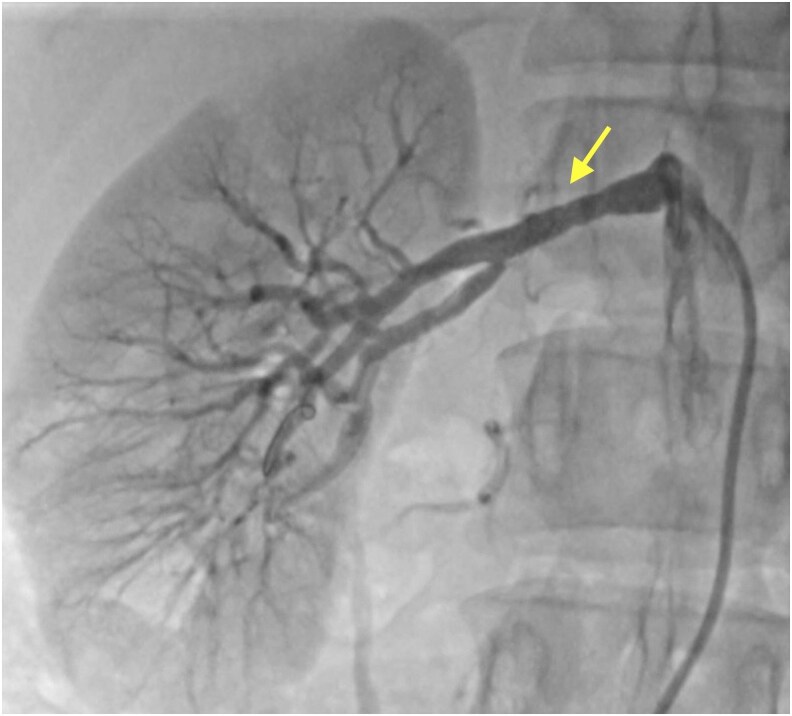
Post-revascularization appearance of the right renal artery following stent placement.

The occluded segment of the right renal artery was successfully crossed using a hydrophilic guidewire. Sequential predilation was performed with appropriately sized balloons, followed by implantation of a drug-eluting stent, resulting in optimal restoration of vessel patency (*Figure 4*; see Video  *S3*). Post-procedurally, the patient’s blood pressure became well controlled, allowing down-titration of antihypertensive therapy to perindopril 5 mg/day and amlodipine 5 mg/day. During hospitalization and subsequent home monitoring, blood pressure readings remained below 120 mmHg systolic and 80 mmHg diastolic on this regimen. To ensure long-term stent patency and protect against atherosclerotic cardiovascular disease, aspirin 100 mg and atorvastatin 40 mg were added to the patient's treatment. A marked improvement in renal function was observed 2 weeks after the procedure. Follow-up laboratory tests revealed a urea level of 60 mg/dL, a serum creatinine level of 1.27 mg/dL (within the normal reference range for adult women), and an estimated glomerular filtration rate of 62 mL/min/1.73 m2, indicating significant improvement compared with baseline values. Following the procedure, the patient has been followed on an outpatient basis for 4 years with 6-month intervals. At 4-year follow-up, blood pressure remained controlled on perindopril 5 mg/day and amlodipine 5 mg/day. Both office and home blood pressure measurements have remained well controlled, and renal function tests have shown a stable course.

## Discussion

RAS is a well-recognized cause of ischaemic nephropathy and secondary hypertension; however, CTO of the renal artery represents a much less frequent and advanced manifestation, accounting for approximately 5–10% of cases among patients with RAS and occurring only rarely in younger individuals, in whom available evidence is largely limited to isolated case reports.^3,4^ Medical therapy is recommended as the first-line treatment for RAS. Optimal management emphasizes strict blood pressure control, with preferential use of renin–angiotensin–aldosterone system blockers, particularly in unilateral disease. In addition, statin therapy and aspirin are recommended to slow disease progression.^3,4^ Large randomized clinical trials have demonstrated that renal artery revascularization does not significantly improve mortality, blood pressure control, or progression of renal dysfunction compared with medical therapy alone.^3,4^

However, these trials largely excluded high-risk patient populations with RAS. Patients presenting with refractory or accelerated hypertension despite optimal medical therapy, recurrent pulmonary oedema, or rapidly progressive deterioration of renal function represent a high-risk subgroup in whom medical therapy alone is frequently insufficient and clinical outcomes remain poor.^5–7^

In this context, renal artery revascularization may be considered in carefully selected high-risk patients when irreversible parenchymal damage has not yet occurred. This consideration is particularly relevant in chronic total renal artery occlusion, where therapeutic decision-making should be guided by markers of renal viability rather than angiographic severity alone. Preserved renal size, demonstrable viable parenchyma on DMSA scintigraphy, and the presence of well-developed collateral arterial supply are key indicators of potential reversibility and functional recovery following revascularization.^8,9^ Beyond serving as a marker of renal viability, collateral circulation in renal artery CTO plays a pivotal mechanistic role by maintaining residual renal perfusion, limiting ischaemic injury, and preventing irreversible parenchymal fibrosis. This sustained perfusion preserves functional renal tissue and supports the potential for meaningful recovery after restoration of antegrade flow. In contrast, the absence of effective collateralization, renal atrophy, a pole-to-pole renal length below 8 cm, or diffuse cortical scarring is associated with a low likelihood of benefit, favouring continued medical therapy. The present case underscores the clinical importance of collateral arterial support and meticulous patient selection in identifying individuals with renal artery (CTO) who may derive sustained benefit from revascularization.

## Conclusion

CTO of the renal artery represents an uncommon but clinically significant cause of resistant hypertension and renal dysfunction. Although large randomized trials have shown neutral results for renal artery revascularization in unselected populations, this case illustrates that carefully selected high-risk patients may still derive meaningful benefit. In particular, preserved renal size, demonstrable parenchymal viability, and well-developed collateral arterial supply appear to be key determinants of reversibility in renal artery CTO. These findings emphasize the importance of timely diagnosis, meticulous patient selection, and individualized therapeutic decision-making when considering revascularization in this rare but challenging clinical setting.

## Supplementary Material

ytag373_Supplementary_Data

## Data Availability

The data supporting the findings of this case report are available and can be shared upon reasonable request from the corresponding author. All data have been anonymized to protect patient confidentiality.

## Lead author biography

Doğancan Ceneli, MD, was born on July 18, 1993, in Manisa, Turkey. He graduated from Kocaeli University Faculty of Medicine (2011–2017) and completed his cardiology residency training at Kartal Koşuyolu High Specialization Training and Research Hospital. After working as a cardiology specialist at Marmara University Faculty of Medicine, Pendik Training and Research Hospital between 2023 and 2025, he has been practicing as a cardiology specialist at Kartal Koşuyolu High Specialization Training and Research Hospital since September 2025.

## Notes

**Consent:** The authors confirm that written informed consent for the submission and publication of this case report, including images and associated text, was obtained from the patient in accordance with COPE guidance.
